# Highly Versatile Cloud-Based Automation Solution for the Remote Design and Execution of Experiment Protocols during the COVID-19 Pandemic

**DOI:** 10.1177/2472630320971218

**Published:** 2020-11-19

**Authors:** Piero Zucchelli, Giorgio Horak, Nigel Skinner

**Affiliations:** 1 Andrew Alliance SA, Vernier, GE, Switzerland

**Keywords:** pipetting robot, lab automation, connected lab, cloud-based laboratory software

## Abstract

There is an urgent need to accelerate the development and validation of both diagnostics and vaccines for COVID-19. These priorities are challenging both public and private sector research groups around the world and have shone a spotlight on both existing bottlenecks in the research workflows involved as well as on the implications of having to do much of this work remotely because of enforced social distancing and lockdown measures. The ability to respond quickly to rapidly evolving events, coupled with an emerging understanding of the disease and its pathology, as well as different mutations of the virus, necessitates a highly flexible liquid-handling automation solution that is amenable to rapid switching between different assay workflows and processes to be exploited tactically as needed. In addition, the use of cloud-based software imparts a unique benefit in enabling multiple research groups and remote technical staff around the world to have ready access to the same protocols in real-time without delays, down to the required level of detail, sharing methods and data (for example, in faster clinical trials). Informed by a recent use case, this article explores these issues alongside the recent development and deployment of an automation solution, whose unique approach in terms of both its cloud-native software and its highly modular hardware aligns especially well with achieving the challenge set by this new frontier in the bioanalytical laboratory.

## Resumen

Existe una necesidad urgente de acelerar el desarrollo y la validación tanto de diagnósticos como de vacunas para COVID-19. Estas prioridades son un desafío para los grupos de investigación del sector público y privado en todo el mundo y han puesto de relieve los cuellos de botella existentes en los flujos de trabajo de investigación involucrados, así como las implicaciones de tener que hacer gran parte de este trabajo de forma remota debido al distanciamiento social y el bloqueo forzados medidas. La capacidad de responder rápidamente a eventos que evolucionan rápidamente, junto con una comprensión emergente de la enfermedad y su patología, así como las diferentes mutaciones del virus, requiere una solución de automatización de manejo de líquidos altamente flexible que se pueda cambiar rápidamente entre diferentes flujos de trabajo de análisis. y procesos que se explotarán tácticamente según sea necesario. Además, el uso de software basado en la nube brinda un beneficio único al permitir que múltiples grupos de investigación y personal técnico remoto de todo el mundo tengan acceso inmediato a los mismos protocolos en tiempo real sin demoras, hasta el nivel de detalle requerido, compartiendo métodos y datos (por ejemplo, en ensayos clínicos más rápidos). Basado en un caso de uso reciente, este artículo explora estos problemas junto con el desarrollo y la implementación recientes de una solución de automatización, cuyo enfoque único en términos de su software nativo de la nube y su hardware altamente modular se alinea especialmente bien con el logro del desafío establecido por este nueva frontera en el laboratorio bioanalítico.

## Abstrait

Il est urgent d’accélérer le développement et la validation des diagnostics et des vaccins contre le COVID-19. Ces priorités mettent au défi les groupes de recherche des secteurs public et privé du monde entier et ont mis en lumière à la fois les goulots d’étranglement existants dans les flux de travail de recherche impliqués ainsi que les implications de devoir faire une grande partie de ce travail à distance en raison de la distanciation sociale et du verrouillage. les mesures. La capacité de réagir rapidement à des événements en évolution rapide, associée à une compréhension émergente de la maladie et de sa pathologie, ainsi qu’aux différentes mutations du virus, nécessite une solution d’automatisation de la manipulation des liquides très flexible qui se prête à une commutation rapide entre les différents flux de travail de test. et les processus à exploiter tactiquement au besoin. En outre, l’utilisation de logiciels basés sur le cloud confère un avantage unique en permettant à plusieurs groupes de recherche et personnel technique à distance du monde entier d’avoir un accès facile aux mêmes protocoles en temps réel sans délai, jusqu’au niveau de détail requis, en partageant méthodes et données (par exemple, dans des essais cliniques plus rapides). Informé par un cas d’utilisation récent, cet article explore ces problèmes parallèlement au développement et au déploiement récents d’une solution d’automatisation, dont l’approche unique en termes à la fois de son logiciel cloud natif et de son matériel hautement modulaire s’aligne particulièrement bien sur la réalisation du défi posé par cette solution. nouvelle frontière dans le laboratoire de bioanalyse.

## 抽象

迫切需要加快对COVID-19的诊断和疫苗的开发和验证。这些优先事项正挑战着世界各地的公共和私营部门研究小组，并且聚焦于所涉及的研究工作流程中的现有瓶颈，以及由于强制性的社会疏远和封锁而不得不远程进行大量这项工作的影响措施。快速响应快速发展的事件的能力，以及对疾病及其病理学以及病毒不同突变的新认识，需要高度灵活的液体处理自动化解决方案，该解决方案必须能够在不同的检测工作流程之间快速切换以及需要在战术上加以利用的流程。此外，基于云的软件的使用为世界各地的多个研究小组和远程技术人员提供了独特的优势，使他们可以实时，实时地访问同一协议，而无需延迟，直至达到所需的详细程度，共享方法和数据（例如，在更快的临床试验中）。鉴于最近的一个使用案例，本文探讨了这些问题以及自动化解决方案的最新开发和部署，该解决方案在其云原生软件和高度模块化的硬件方面的独特方法尤其适合于解决由此带来的挑战生物分析实验室的新领域。

## 概要

COVID-19の診断とワクチンの両方の開発と検証を加速する緊急の必要性があります。これらの優先事項は、世界中の公共部門と民間部門の両方の研究グループに挑戦しており、関連する研究ワークフローの既存のボトルネックと、強制的な社会的距離と封鎖のためにこの作業の多くをリモートで実行する必要があることの影響にスポットライトを当てています。対策。急速に進化するイベントに迅速に対応する能力と、疾患とその病理学、およびウイルスのさまざまな変異に関する新たな理解が相まって、さまざまなアッセイワークフロー間の迅速な切り替えに適した柔軟性の高い液体処理自動化ソリューションが必要です。必要に応じて戦術的に活用されるプロセス。さらに、クラウドベースのソフトウェアを使用すると、世界中の複数の研究グループやリモートの技術スタッフが、必要な詳細レベルまで、遅延なくリアルタイムで同じプロトコルにすぐにアクセスできるという独自のメリットが得られます。方法とデータ（たとえば、より高速な臨床試験）。この記事では、最近のユースケースに基づいて、自動化ソリューションの最近の開発と展開とともにこれらの問題を探ります。このソリューションのクラウドネイティブソフトウェアと高度にモジュール化されたハードウェアの両方に関する独自のアプローチは、これによって設定された課題の達成と特によく一致します。生物分析研究所の新しいフロンティア。

## Introduction

We are facing a human crisis unlike any we have experienced. . .our social fabric and cohesion is under stress.

—*UN Deputy Secretary General Amina J. Mohammed during a digital meeting of the Forum’s COVID Action Platform on April 8, 2020*

On December 31, 2019, Chinese authorities alerted the World Health Organization (WHO) of pneumonia cases in Wuhan City, Hubei province, China, with an unknown cause. The outbreak was declared a Public Health Emergency of International Concern on January 30, 2020, and on February 11, 2020, WHO announced a name for the new coronavirus disease: COVID-19.

From Asia to Europe and further West to the United States of America, countries went into various states of lockdown, some faster than others, according to differing local scientific advice and policy. Wuhan City along with other cities in Hubei Province, went into lockdown on January 23, and other leading Western economies followed as the virus rapidly spread West, resulting in widespread distribution of the disease by late June 2020.^1^

On March 15, Germany closed its borders to Austria, Denmark, France, Luxembourg, and Switzerland. By March 22, curfews were imposed in 6 German states, whereas other states prohibited physical contact with more than 1 person who resides outside of a household.

On the March 20, California ordered its 40 million residents to stay at home unless required to make essential trips, becoming the first US state to implement such extreme measures in a bid to contain the rapidly expanding coronavirus outbreak.

Social distancing measures meant that over a short period of time, companies in the world’s leading economies needed to rapidly pivot to a situation in which most of their employees were required to work remotely, from home, in some cases leaving a small critical skeleton staff in company facilities. In addition, to weather the economic storm, companies also put staff on furlough to minimize expenditure.

This of course included pharmaceutical and clinical diagnostic companies, together with academic and government research labs, all being critical to the urgently required development of both diagnostic tests for both the SARS-CoV-2 virus and the presence of antibodies to it and for a vaccine against the virus.

On the importance of testing:

We have a simple message for all countries: test, test, test. Test every suspected case. (UN Deputy Secretary General Amina J. Mohammed, at the media briefing on March 16, 2020)

On the need to find a vaccine:

This infection is not going to disappear . . . without science leading us to vaccines, we will get second and third waves of this. . . . Unless we do produce drugs and vaccines we are not going to have an exit strategy. (Jeremy Farrar, Director of the Wellcome Trust, during a digital meeting of the Forum’s COVID Action Platform on April 8, 2020)

Given that, from late March, the vast majority of leading research labs, both private and public sector, were working remotely, the need to accelerate development of both rapid diagnostic kits and vaccines had become uniquely challenging. It is important to note that under normal circumstances, required research and development (R&D) work would be highly time-consuming and resource intensive.

The COVID-19 outbreak has had a major impact on clinical microbiology laboratories in the past several months. Real-time reverse transcriptase polymerase chain reaction (RT-PCR) assays remain the molecular test of choice for the etiologic diagnosis of SARS-CoV-2 infection, whereas antibody-based techniques are used as supplemental tools. PCR-based tests need to be conducted in a suitably equipped laboratory by experienced analysts and take time, whereas a serological test, such as the Cerascreen corona virus antibody test, could involve the sample being collected at home and sent to a certified medical laboratory to be tested for SARS-CoV-2 antibodies, with results being reported within 12 to 48 h of receipt of the sample. Accurate diagnosis requires the use of both types of test, and the scaling of such testing is essential to the rapid, accurate diagnosis and monitoring of SARS-CoV-2 infections, greatly assisting in the control of this outbreak.

The development of a new vaccine can take more than 10 y, involving multiple stages: exploratory, preclinical, clinical trials, regulatory review and approval, manufacturing, and quality control. Being a highly coordinated scientific activity, its acceleration cannot be achieved by simply putting more resources into the game: new tools and new modus operandi are required.

The increasing need to accelerate research, increase the productivity of R&D, and improve return on investment has resulted in an insatiable appetite for the increasing use of automation, especially in the vertical ontology of liquid handling, which is often considered a critical bottleneck, both because of throughput as well as to repeatability. The benefit of automation holds true in the development of diagnostic kits and vaccines for COVID-19 but with the singular difference of needing to operate with a workforce that is distributed across multiple remote locations, while being capable of easy reconfiguration due to rapidly evolving circumstance, such as the ongoing effort to better understand the mechanistic nature of this disease.

## Discussion

### The Current State of Automation

The automation of processes such as liquid handling and sample management has been increasingly adopted by the pharmaceutical industry, in particular, over the past few decades and, in parallel, in clinical diagnostics labs as well. This has been partly driven by the need to scale throughput and process samples for applications such as high-throughput screening more quickly, partly to ensure that highly qualified, well-paid, scientific staff are focused on high-level activities (vs. low-level, laborious, repetitive work) and partly to address the challenge posed by the reproducibility crisis, in which it has been estimated that of 238 published scientific articles, barely 46% could be reproduced,^1^ and the claim that replication efforts for only 2 of 5 cancer papers have been successful.^2^

It is revealing to note that “. . .more than 70% of researchers have failed to reproduce a colleague’s experiment and more than 50% have failed to reproduce their own.”^3^ More than 35% of irreproducibility has been attributed to manual errors in the performance of experiments and data reporting.^4^

A number of studies conducted in recent years have revealed alarmingly low levels of reproducibility, especially within the life sciences. These range from drug discovery^5^ to psychology,^6^ including synthetic biology^7^ and medical research.^8–10^ One study estimated that the costs of having to repeat experimental work were as high as $28 billion.^11^ In the context of a pandemic situation, the presence of unreliable and irreproducible information is an even larger problem, not allowing sufficient time to check and revalidate hypotheses that may lead to incorrect strategies, with possible direct consequences on human lives.

There has been considerable debate about the causes, although common factors include basic flaws in experimental practice,^4,12,13^ variability in antibody-based methods and assays,^14^ and article retractions reported in PubMed. Articles were reported to have been retracted because of procedural errors in analytical workflows together with contamination, for example, in sequencing and cloning, which accounted for many of these, with analytical errors noted to be on the rise.^15^ Contamination has further been identified as a major source of errors in large sequencing studies.^16^ Of particular importance is the ability to define unambiguously the initial conditions prevailing when the process was performed.^17^

Perhaps most critically is the way in which research is actually disseminated and reproduced.

The process of scientific publication and dissemination is highly dependent on the use of natural language (usually English) and the lack of precision associated with both the writing of protocol and its interpretation by a person whose mother language may be different, as well as the use of humans for execution of laboratory work and much of the potential variability associated with that.

Manual pipetting has also been identified as an important source of error, with errors often attributed to any one of a number of factors:

- Viscosity of samples: this should determine the type of pipetting applied (e.g., reverse pipetting for viscous samples, etc.)
- Prewetting pipette tips: failure to do so can lead to liquid loss in the pipette tip due to evaporation
- Temperature of samples: sample volume can be altered if the pipette and the liquid being dispensed are not temperature equilibrated
- Degree angle of pipetting (aspirate at 90° and dispense at 45°)
- Working too quickly: after aspirating, failure to pause with the pipette tip in the liquid can lead to underdelivery, because the liquid is not still at first insertion and requires about 1 s to settle
- Using the wrong pipette tips: failure to choose the proper tips for a given type of pipette can lead to an inadequate seal between the pipette and tip, causing leakage and sample loss
- Lack of adequate training on the correct use and storage of manual pipettes
- Decrease in pipetting performance throughout the day due to operator fatigue

For example, in drug discovery, IC_50_ assays, commonly used to evaluate drug efficacy, and assay development procedures as well as standard-curve generation involve the serial dilution of compounds, proteins, or detection agents and can be potential casualties of manual pipetting errors. These processes can be streamlined by using automated liquid-handling equipment with serial dilution capabilities, addressing two common workflow challenges: error propagation across the columns or rows of a microtiter plate due to transfer inaccuracies that lead to less accurate and less precise dispensing and the risk of error in the calculation of serial dilutions themselves.

When looking at the automation solutions available, a scale of liquid-handling automation solutions can be observed, ranging from automating the programming of electronic pipettes (semiautomated solutions, if you like), through mid-range liquid-handling equipment to more costly, self-contained, and fully integrated automation platforms. This latter category has its origins in the initial drive to significantly scale the throughput of large sample batches for areas such as high-throughput screening in drug discovery.

Growth in the low- to medium-range liquid handlers partly came down to lower throughput requirements demanding lower cost solutions and partly due to an increased number of companies acquiring automation solutions to address workflow challenges with their own (often analytical products) experienced by users in areas such as sample preparation.

The preparation of sample and reagent for many analytical workflows often involves the complicated manipulation and handling of different reagents, frequently precious tissue and serum samples, and potentially complex actions (ontologies) being carried out such as shaking, heating/cooling, magnetic bead separation, weighing, and solid-phase extraction (for liquid chromatography/mass spectrometry; LC/MS), with a variety of different labware such as 96- and 384-well microtiter plates and different sizes of tubes and columns. Moreover, the management of such automation has demanded sophisticated software and investment in personnel experienced in being able to program in languages such as C++ and Python or just complex instrument software (particularly where workflow scheduling is required), making such technology relatively inaccessible to the biologist whose standard training often does not include such skills. The communication between humans with different backgrounds unavoidably adds to the uncertainty in the correct execution of a process.

### The Connected Lab: Benefits of “The Cloud”

Pharmaceutical research has become far more distributed over the past decade in part due to increased collaboration between drug discovery groups from different sites, organizations, and companies and more recently of course driven by necessity due to lockdown measures instituted during the COVID-19 pandemic. In some cases, virtual or semi-virtual companies are formed by outsourcing all, or a subset of, chemistry and biology services to contract research organizations. Distributing research in these ways can have profound advantages, but managing distributed research has unique challenges. Informatics plays an important part in the drug discovery process. Proper collection, collation, and reporting of results is imperative to show value from a project and provenance to the data. This can become increasingly complicated when compounds and data are being generated on multiple sites and by different organizations.

Driving this informatics revolution, especially over the past decade, there has been an explosion in the data being both generated, captured, and analyzed. This is often referred to as the “Internet of Things” (IoT). In the pharmaceutical industry, this has been the case both for research, driven by the need to better understand disease and develop new therapeutics and (increasingly companion) diagnostics through translational research, and for development, with ever increasing regulatory oversight and the need to understand adverse side effects of drug candidates.

**Figure 1.**
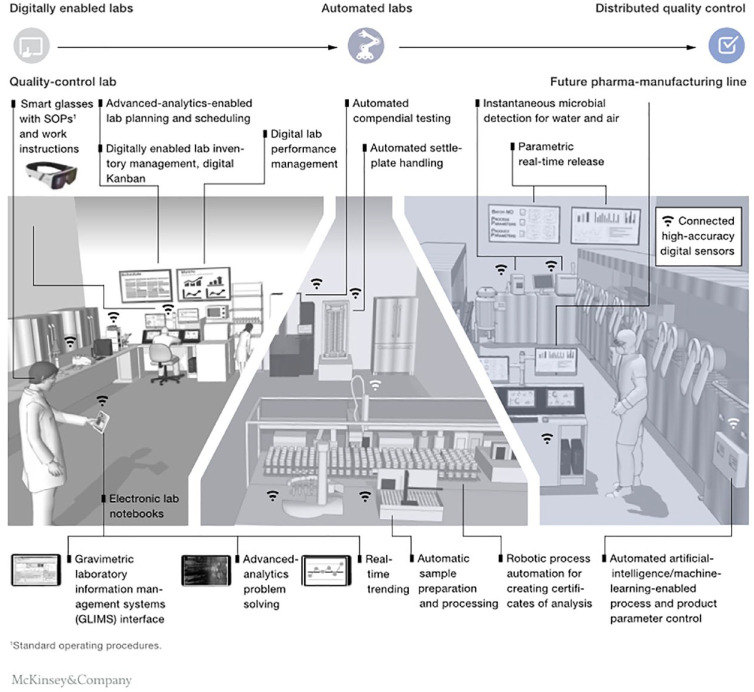
Digitization and automation will transform quality control work in the lab and on the shop floor by introducing new ways of working.^18^

In response to this landscape, many laboratories have already implemented digitalization technologies or are in the process of doing so. But with the rise of Industry 4.0, which brings increased automation and digital information transfer in manufacturing, many organizations are contemplating what this means for the future of the lab. As illustrated in **Figure 1**, Laboratory 4.0 (Lab 4.0) brings these concepts into the lab, automating the capture and flow of digital data from all the disparate networked instruments and systems within the lab. The benefits are manifold, including more accurate results, reduced costs, greater efficiency, and improved collaboration.

Lab 4.0, coupled with IoT, has driven the idea of the “connected lab,” where disparate data sets can be integrated in real time with the very tangible and immediate benefit of improved repeatability and productivity.

Let us put this into perspective using a specific example. Most sample prep workflows will involve the weighing of a reagent, say a lyophilized enzyme powder, and, separately, the pipetting of a buffer to reconstitute the powder. Each instrument will have been calibrated, and each measurement will have been achieved within a set of error bars determined by the method and technique used. A protocol will have been followed, and in today’s lab, each measurement will have been done discretely with little attempt being made to take account for real-time conditions of temperature and humidity, instrument status, or the result of the actual measurement. If we translate this scenario into a connected lab, we could imagine both weighing scale and pipettor being connected to the same software, potentially hosted in the cloud, which is able to optimize one operation based on the results and local conditions of the other. In this way, we benefit from further improvements in repeatability and reduce the uncertainty associated with the data output of a particular analytical workflow such as an enzymatic assay.

This ability to be smart about the execution of a particular workflow by real-time data gathering alongside learning and adjustment of subsequent experimental parameters is one benefit, but there are others, such as the ability to potentially trace all steps taken in the workflow and knowing what particular manipulation was done, when it was done, where it was done, and by whom.

This could be helpful if trying to track down the source of DNA contamination in a next-generation sequencing or quantitative polymerase chain reaction (qPCR) protocol. It could also be useful for regulatory purposes, when looking to verify testing done on a new drug compound. This same benefit, of course, also serves to mitigate liquid-handling errors occurring in serological assays and reverse RT-PCR (rRT-PCR) preps, which are the assay types currently in development in labs worldwide in response to COVID-19. In the case of rRT-PCR, workflows are highly sensitive to any departure from standard operating procedures. Obtaining a clean, successful PCR requires samples free of exogenous DNA. A lab can be a source of contamination from previously amplified products or the user’s own DNA. Mitigating measures include designating and using distinct areas for sample preparation, PCR setup, and post-PCR analysis and carefully differentiating between operations conducted in the pre- and post-PCR areas. The traceability from cloud software conceived for the design of laboratory methods and the execution of the corresponding experiments can provide the necessary a posteriori information about protocol followed, times, volumes aliquoted, samples, reagents, equipment, and operator identification.

### Cloud-Based Automated Lab Systems for Infectious Diseases

More generally, a cloud-based automated system may refer to the establishment of information systems for effective disease monitoring, risk assessment, and early warning management for international disease outbreaks. In this instance, a cloud computing framework can effectively provide the required hardware resources and information access and exchange to conveniently connect information related to infectious diseases and develop a cross-system surveillance and control system for infectious diseases of pandemic proportion.

However, this is not the type of cloud-based system discussed in this article, as is perhaps evident from the previous section, its emphasis on the value of Lab 4.0, and its reference to ontologies such as pipetting and weighing. Here we focus on a cloud-based system focused on lab automation, whose benefit to infectious disease research relates to the simplification and greater reliability of lab workflows, such as liquid handling, which is core to assay development, whether for diagnostic or therapeutic development. In turn, developers of diagnostic kits and therapeutics can reduce experiment turnaround time, scale sample throughput, share protocols order to collaborate more easily, and potentially gain from full traceability, whether for protocol troubleshooting, sample tracking, or regulatory compliance.

Companies such as Tecan use cloud-based services (Introspect and Common Notification System) for monitoring, reporting, and analysis within lab automation platforms such as their Fluent and Freedom EVO liquid handlers. Although a highly valid use of the cloud, this article argues that the real value of cloud-based software lies in its ability to address the errors inherent in protocol design, execution, and replication, as highlighted in various investigations into the reproducibility crisis.^3^ In this regard, it is not therefore surprising that Tecan is partnering with companies such as Synthace^19^ to deepen the integration between Synthace’s Antha software and Tecan’s Te-Chrom automated chromatography system, eliminating manual processing steps for customers using RoboColumn systems and other compatible consumables.

Synthace developed a cloud-based software called Antha. Antha was perhaps the first bona fide attempt to create a high-level protocol language for general purpose computation in biology. Built atop Google’s Go language,^20^ Antha is an open-source high-level language that combines a complete, fully featured programming language with a number of domain-specific features such as liquid-handling planning not only to allow specification of the most complex manual protocols but also to incorporate sophisticated logic and algorithms within protocols, enabling experiments of an entirely new level of complexity to be defined. The Antha language is the central component of AnthaOS, a service-oriented architecture providing device integration, experimental logging, stock management, and network interfaces for external code and services. Protocols written in Antha are executed on automation platforms developed by other companies. Synthace’s goal is to create a universal language for biology, facilitating the consistent execution of different ontologies with experiment workflows. Antha is platform agnostic and so can work with different liquid-handling instrumentation, and the protocols are easily shareable. It also has the added benefit of enabling full design-of-experiments functionality. All instrument methods, however, would still need to be created within their own respective software environments.

Cloud-based software is often referred to as software as a service (SaaS). Saas is a licensing and delivery model in which software is licensed on a subscription basis and is centrally hosted on the cloud. That said, it should be noted that because of concerns over data security, large pharmaceutical firms tend to have such software installed on a server on site rather than accessing the cloud-based version.

Software enabling the design and execution of protocols on liquid-handling automation does not, of course, need to be cloud based. **Table 1** compares some key providers. One of the differentiating features of offerings in this area is whether the company providing the software also provides the automation hardware or whether that is, in fact, provided by other/partner companies. Integra, Opentrons, and Andrew Alliance are all established suppliers of liquid-handling automation, whereas Synthace, Tetrascience, Benchling, Labforward, and Riffyn are not hardware suppliers and focus solely on software.

**Table 1.** Comparison of Software Platforms.

	Integra (VIALAB^a^)	Opentrons^a^	Synthace	Tetrascience	Benchling	Labforward	Riffyn	Andrew Alliance (OneLab)
Using own (closed)/other (open) automated solution	Closed	Closed	Open	Open	Open	Open	Open	Device-agnostic protocols but only uses own automation
Ease of use (UX)	Easy to use	Easy to use	Easy to use once user has completed a few days of training	Easy to use once user has completed a few days of training	Easy to use once user has completed a few days of training	Easy to use	Easy to use once user has completed a few days of training	Easy to use
Ease of accessibility	Plug and play	Plug and play	Requires installation and customization	Requires installation and customization	Requires installation and customization	Requires installation and customization	Requires installation and customization	Plug and play
Automated solution	Liquid handling	Liquid handling and other small connected devices	Liquid handling, analytical instrument, and bioreactor	All connected devices; no information on liquid handler	Liquid handling and analytical instrument	All connected devices; no information on liquid handler	Analytical instruments	Liquid handling and other small connected devices
Protocol/workflow creation	Yes, but solely pipetting	Yes	Yes	No	No information	Yes	Yes	Yes
Visual guidance of an experiment	No, only automation	No, only automation	No, only automation	No information	No, only automation	Yes	Yes	Yes
Data collection and visualization	No	No	Yes	Yes	Yes	Yes	Yes	No

a Not cloud based.

It should be noted that neither Integra’s software (VIALAB) nor that of Opentrons are cloud based, and they are included here for the purpose of comparison of cloud-based software for liquid-handling automation versus non–cloud based. The significance of this comparison with regard to COVID-19 in particular is important. As discussed in the introduction, the COVID-19 took the global research community by surprise, demanding rapid development of diagnostics and therapeutics during times of intense workforce restriction (lockdown, remote working, illness, furlough). Rapid R&D demands considerable flexibility at the workflow level, for example, for assay development and optimization. This implies being able to easily use different vendor labware, scale from using tubes to microplates, adapt ontologies, and, of course, quickly and accurately create, execute, and share protocols, in many cases, remotely! These requirements place demands on both software and hardware. Put another way, an ideal solution would mate a versatile cloud-based software with highly adaptable liquid-handling automation.

**Table 1** (below) also compares these different companies in terms of their ease of use, ease of accessibility, extent of workflow automation, whether protocols can be created or not, visual guidance of an experiment, and data collection and visualization.

Opentrons, through its OT-2 liquid-handling platform, provides a low-cost solution, and although it has made available a number of commonly used protocols, the development of new ones would require that users either work with Opentrons directly or develop a new protocol themselves, albeit demanding skills in Python programming, which is infrequent in the life sciences community. Its relatively small footprint limits the range of different ontologies that can be used. Integra developed its VIALAB software for its Assist Plus and range of electronic pipettes. Users can develop their own protocols with the software, albeit with a few hours of training. The liquid-handling automation has the innovative feature of variable tip spacing facilitating tube-to-plate transfers and plate reformatting.

Tetrascience provides a cloud-based software that gathers data and connects and monitors devices for fleet management purposes rather than for the creation and sharing of experiment protocols. Labforward has developed a powerful platform that is used for data gathering and the connection and monitoring of a wide range of digital devices in the laboratory. Notably, it has sample inventory capability and can integrate with electronic laboratory notebook (ELN)/laboratory information management system (LIM)S. Benchlings offers a similar cloud-based product albeit with integrated sample tracking using a barcode reader, which is especially useful for diagnostic labs. Riffyn’s cloud-based software is used only for data gathering and data visualization purposes and not for designing and executing protocols on liquid-handling automation.

Although not covered in **Table 1**, Thermo Fisher Connect is also a cloud-based platform, enabling users to select from a wide range of protocols and run these on its Thermo E1-ClipTip Bluetooth electronic pipettes. In this case, these would be available for use only on Thermo Fisher products. Thermo also offers Platform for Science, which enables protocol customization and is open access (vs. closed, as is the case with Thermo Fisher Connect), and can be readily integrated with an ELN/LIMS/scientific data management system.

Gilson, a leading manufacturer of pipettes, has a cloud-connected platform (Gilson CONNECT) that connects TRACKMAN Connected (tablet) and PIPETMAN M Connected (pipettes), in which tablet and pipettes interact via Bluetooth, enabling real-time execution and tracking of pipetting protocols. CONNECT is limited in terms of its purvey to Gilson’s own range of electronic pipettes and not fully automated liquid-handling instrumentation.

In considering the relative merits of liquid-handling automation that does or does not use cloud-based software, it is important to recall the benefits of Lab 4.0, as discussed in the previous section, namely, “automating the capture and flow of digital data from all the disparate networked instruments and systems within the lab. The benefits are manifold, including more accurate results, reduced costs, greater efficiency, and improved collaboration.”

It is worth adding that cloud-based approaches are being exploited in different ways in this space. A good example is Transcriptic, whose fully robotic work cells are able to execute entire assay workflows, ensuring that required protocols are executed exactly to specification, minimizing the risk of errors being made.

Andrew Alliance combines both its cloud-based software, OneLab, with its liquid-handling automation, Andrew+, which benefits from being able to combine the benefits of a cloud-native software with the adaptability of its pipetting robot. As such, the remainder of the article describes both the OneLab and the Andrew+ pipetting robot in more detail, illustrating their application to COVID-19 through a use case.

### OneLab: Cloud-Native Design, Execution, and Protocol Sharing

*No one is as deaf as the man who will not listen. (Proverb)*

In Andrew Alliance, the development of a cloud software conceived for the design of laboratory methods and the execution of experiments (later called OneLab) was rooted in listening to users of the original award-winning Andrew pipetting robot between 2013 and 2018, consistent with the company’s underlying philosophy of lean development.^21^

The company’s mission is to “advance science by working with scientists to create a new class of intelligent software, easy-to-use robots, and connected devices that take repeatability, performance and efficiency of laboratory workflows to the level required by 21st century biology” (www.andrewalliance.com/about).

In the life science industry, software has tended to be associated with a poor user experience. In automation, it typically requires programming expertise, and that is assuming that it is possible to create new protocols independent of the vendor.

Design inputs for OneLab, based upon user input told us the following:

- Ease of intuitively designing new protocols, in a simple visual way (drag and drop) without programming knowledge
- Execution of protocols without wires for ease of operation in different environments and disparate locations
- Recording of protocol execution for traceability and audit purposes, including capture of pipette calibration data
- Providing a workspace that is adaptable to the user’s workflow (rather than vice versa)
- Ease of adding new ontologies, tools, and labware so that users are “future proofed”
- Taking full advantage of the IoT to ensure an adaptable solution, ability to optimize according to real-time data and workflow requirements
- Real-time access to technical support

This required a cloud-native software solution, subsequently named “OneLab,” and an evolving ecosystem of connected devices that are programmed by OneLab via an ethernet connection, WiFi, or, for handheld devices, Bluetooth, via an embedded microchip contained in the devices (for example, the Andrew+ pipetting robot or the Pipette+ smart stand), which are automatically paired to the cloud. In fact, the beauty of the concept is that any device in a laboratory could be connected to OneLab provided it has a digital port, through the use of what is referred to as Bridge+, and this is very much the case today for the vast majority of devices used in the lab, including the most mundane, such as the magnetic stirrer.

**Figure 2.**
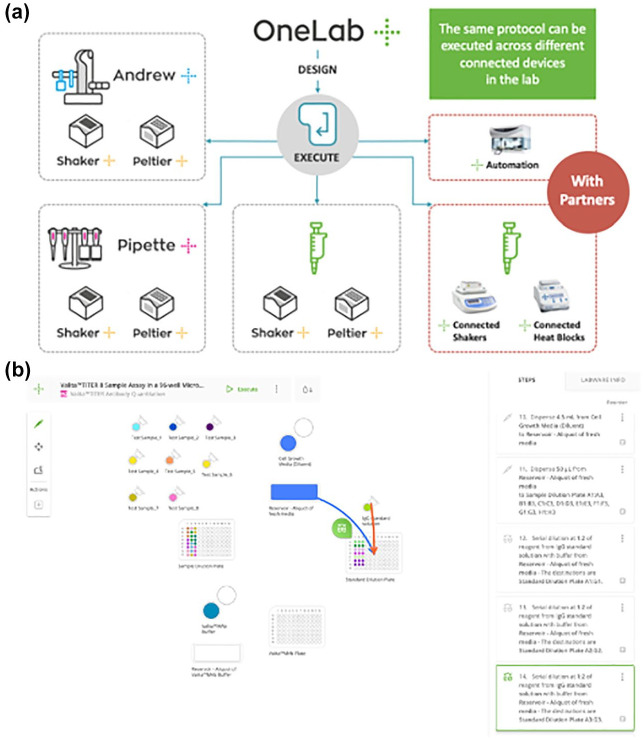
(**a**) Setup options with OneLab. (**b**) Screenshot of OneLab, showing the ValitaTITER protocol.

This conveys a number of distinct advantages. It means that OneLab provides a solution for protocol creation, execution, and dissemination irrespective of whether the user wants to use it for their own existing setup without any connected devices, for guided manual execution, or for a fully automated setup, as illustrated in **Figure 2a**, while providing a vehicle by which partner protocols can be more easily shared with users around the world via the OneLab Online Library, which provides a means of publishing and disseminating validated protocols for ease of adoption by users, enabling them to automate their research without delay.

Partner applications mean that users can gain such access to a partner protocol, such as the ValitaTITER application of Valitacell. ValitaTITER is a fluorescent polarization assay for the detection of immunoglobulin, a critical method for the identification of suitable clones for the manufacture of biopharmaceuticals. This screening process requires accurate and reproducible tools and methods to ensure success.

**Figure 2b** shows a screenshot of the ValitaTITER protocol in OneLab, which gives an idea of the highly intuitive visual layout of this online lab, with editing tools on the left-hand side of the screen for the selection of labware and actions to conduct on that labware; the labware itself together with pipetting actions, highlighted with orange and blue arrows, from source to destination (in this case, in the wells of a microplate); and step-by-step details of each step in the protocol on the right-hand side of the screen.

As of June 2019, Andrew Alliance’s OneLab connected device ecosystem comprises the following:

- Andrew+ pipetting robot
- Pipette+ guided pipetting system
- Shaker+ programmable shaker available for both microplates and tubes
- Peltier+ programmable heater/cooler available for different microplates
- Magnet+ available for Corning Falcon 50 mL centrifuge tubes and PCR plates
- Vacuum+ available for solid-phase extraction for liquid chromatography/mass spectrometry (LC/MS) applications

**Figure 3.**
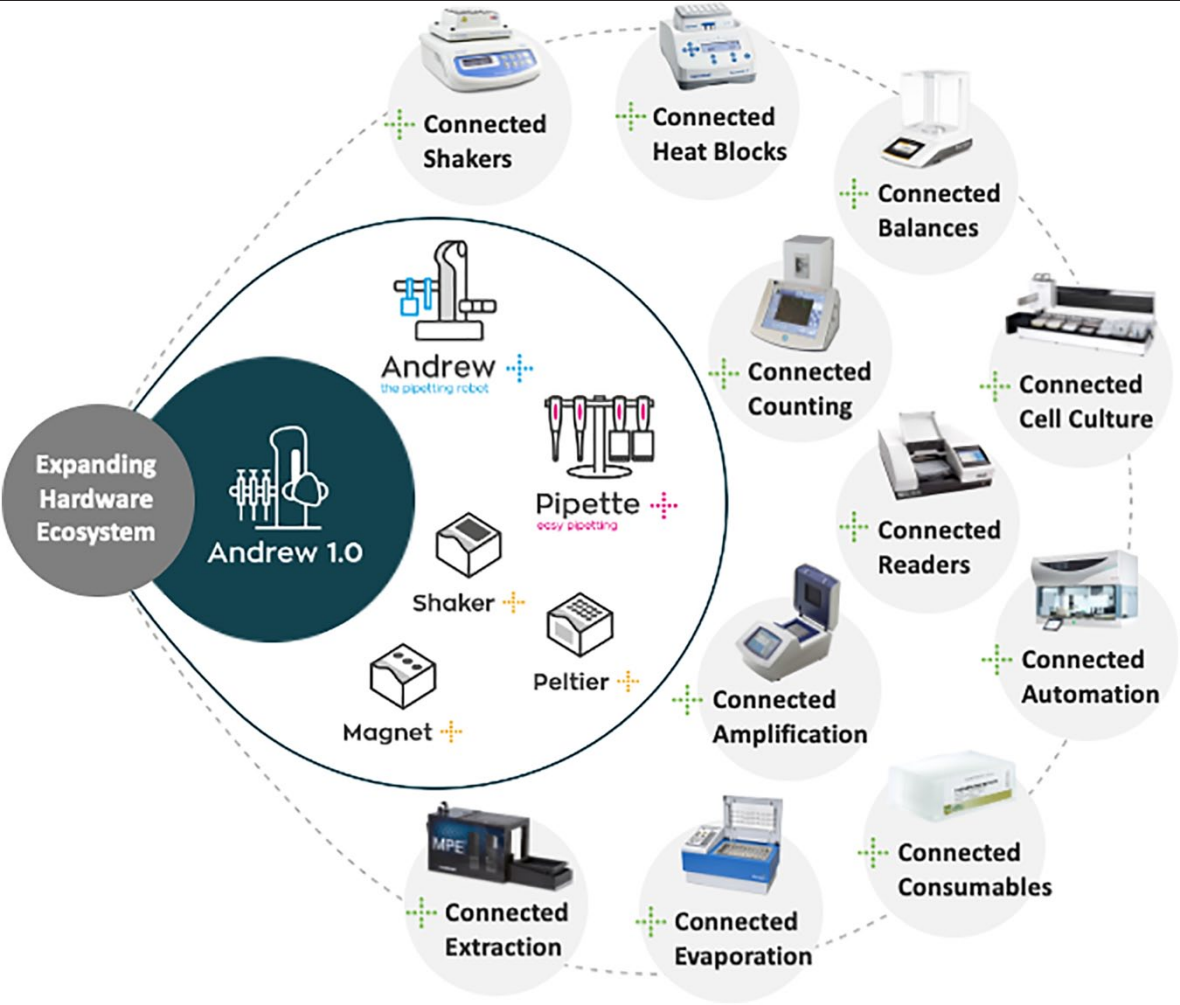
The expanding OneLab ecosystem.

This is illustrated in **Figure 3**, which depicts the evolution of reach of the OneLab software beyond the original AndrewLab software, developed for the Andrew 1.0 pipetting robot. The ability of OneLab to easily incorporate new ontologies makes it especially powerful.

This effectively provides a solution to distributed pharmaceutical research groups and conveniently to those involved in urgent COVID-19 efforts at this time.

**Figure 4.**
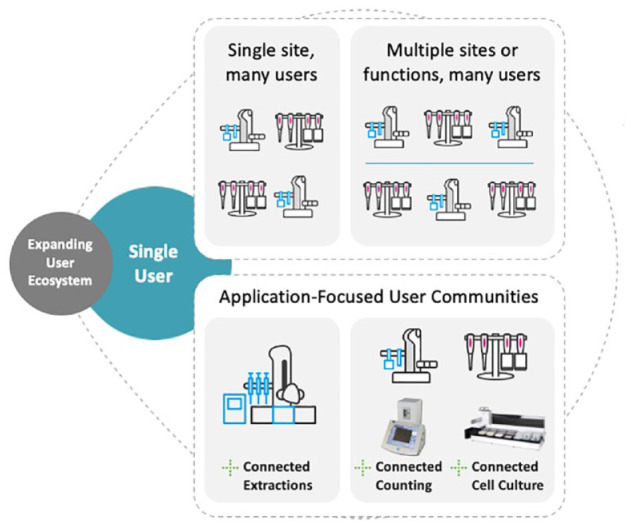
Satisfying the increasingly distributed nature of pharmaceutical research.

More traditional automation solutions have focused on single users. In reality, for a protocol to be successful, it needs to be accessible by multiple users in either the same or disparate, remote locations (**Figure 4**), via an expanding user ecosystem. This is true whether it is for an ongoing drug discovery research project, for a ring trial with multiple labs or a large-scale public-funded project (as in the case of application-focused user communities), for an academic collaboration, or purely for the purpose of quickly and successful repeating the findings of another group’s research.

An ever-increasing range of machine-identifiable consumable holders called “Dominos,” which attach to each other, as well as to the Andrew+ robot, magnetically, enable a wide range of standard labware to be used. The different ontologies (shaking, heating/cooling, magnetic bead separation, micro-elution) can be used interchangeably between Andrew+ and Pipette+, meaning that users can readily use the two together or scale from using Pipette+ to Andrew+.

Although a key driver has been the increasingly dispersed nature of drug discovery research, it has been clear following the onset of the COVID-19 pandemic and ensuing lockdown that the OneLab cloud-native philosophy and ecosystem of connected devices is especially well suited to a situation in which researchers need to conduct experimental work remotely, as has been the case for companies involved in the development of both diagnostic kits to identify the SARS-CoV-2 virus, persons having been infected, and the development of a vaccine.

The use case of Mammoth Biosciences is especially instructive in this regard.

Mammoth Biosciences (South San Francisco, CA) is a leader in CRISPR gene editing and, together with GSK, is developing a rapid diagnostic for the detection of viral RNA associated with the SARS-CoV-2 virus. As part of this effort, they need to optimize the required assay. The challenge is that this work commenced at the time lockdown was announced in the state of California, resulting in the project leader having to work from home.

**Figure 5.**
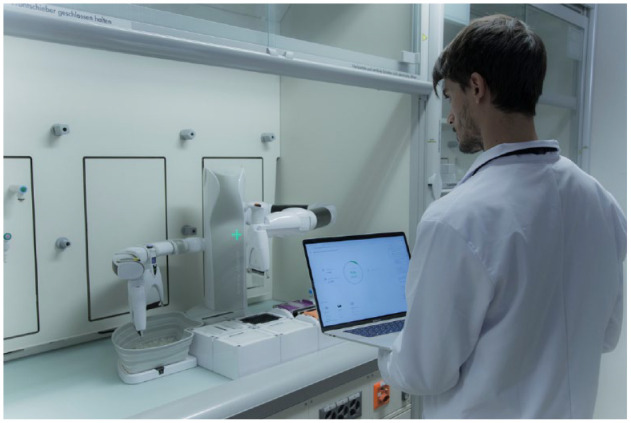
Andrew+ pipetting robot, in a laminar flow hood, being controlled by OneLab.

The company’s existing automation could not be operated remotely, so they required an automation solution that could meet this essential requirement without any knowledge of programming or automation engineering. The Andrew+ pipetting robot (such as that depicted in **Figure 5**) fulfilled this need, in part as it could be operated remotely using OneLab, in part as full traceability was captured, and in part because they were still in the midst of optimizing their assay development protocol and required flexibility in the types of consumables used. Protocols were executed remotely, and the operation of the robot was monitored using a webcam through Zoom.

It is important to note that the system needed to be capable of installation following lockdown. Because the installation is performed remotely within the user’s own “lab,” which is hosted on the cloud, and as both the project leader (working from home) and technical support team (also working from home) are remote, no travel is involved and no social distancing measures are compromised.

Deployment of the OneLab software in the cloud provides maximum functionality, including user management, protocol library, and access to online technical support (referred to as INTERCOM). By default, it also means that the software is automatically updated, which may not be an ideal solution for regulated environments. In this latter regard, the software can be deployed either in a private cloud (EDGE) on the user’s own IT network or onto a standalone server that does not need to be connected to the user’s own network.

### Setting up the Andrew+ Workspace

A point touched upon earlier in this article was that of the ease with which the automation can be adapted to the user’s workflow, rather the more standard requirement for users to have to adapt their workflow to the automation itself. In the middle of a crisis such as COVID-19, with most scientific personnel working remotely, it is clearly not viable to be making changes to a predefined workflow, especially with the urgency demanded of a new diagnostic.

**Figure 6.**
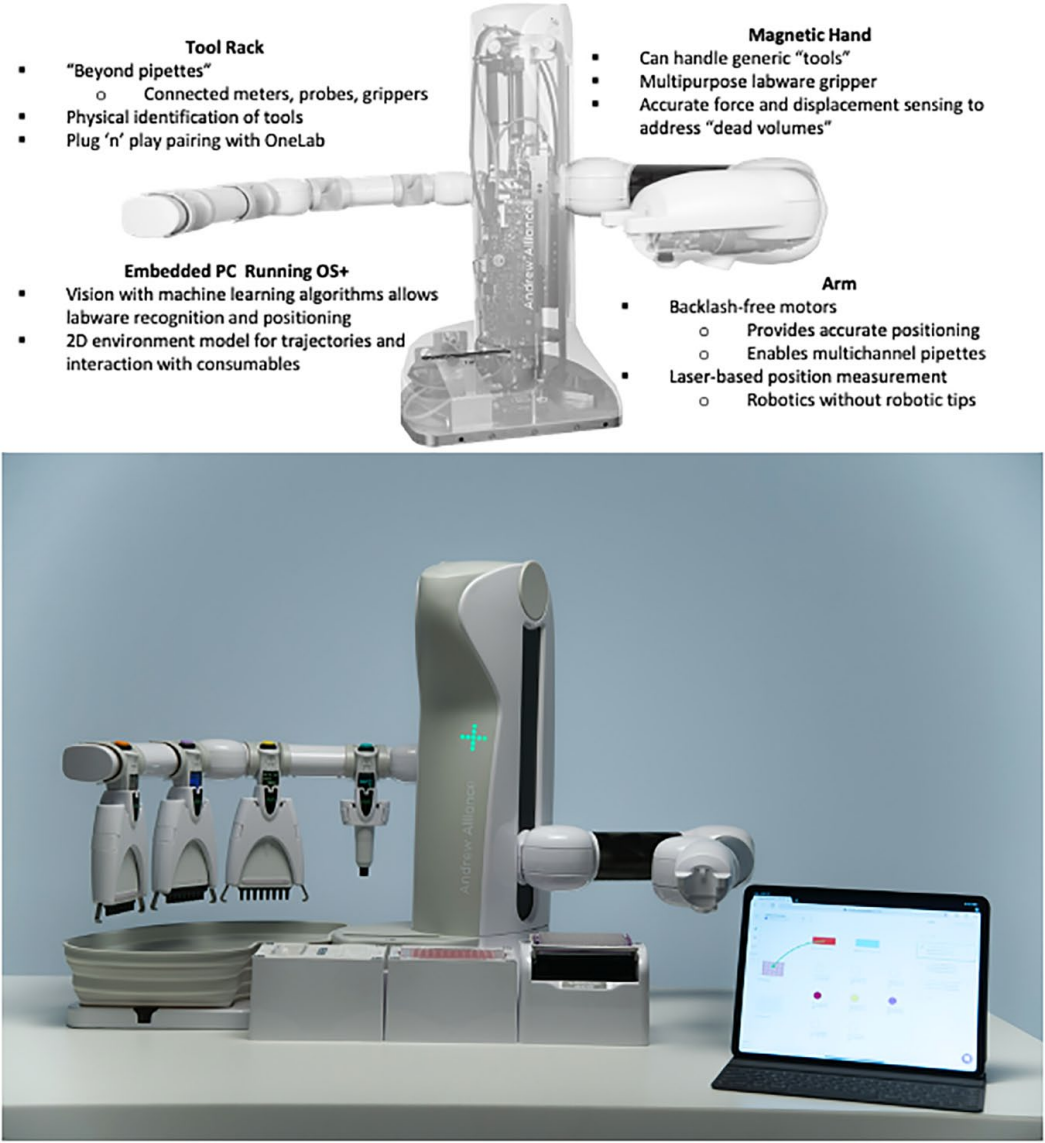
Disposition of Dominos, connected devices, and connected tools on Andrew+.

Most, if not all, automation systems on the market are tooled for a specific application or set of applications, and subsequent changes to user needs invariably involve retooling by the manufacturer, which also typically implies reprogramming of the hardware as well. For this same reason, they also occupy a fixed footprint in the laboratory. This latter point is significant for researchers involved in COVID-19, because much of their work is conducted in biosafety hoods, where automation would need to be operable, quite often alongside other fixed footprint instrumentation. As such, a flexible workspace provides an important advantage (**Fig. 6**).

In the case of the Andrew+ pipetting robot, this flexibility is based on the use of so-called Dominos, as seen in front of the photo of the Andrew+ pipetting robot in **Figure 6**.

Dominos are the components used to interface labware to Andrew+. Each Domino can accommodate a type of labware; for example, a microplate Domino is used for standard ANSI/SLAS plates (6, 24, 48, 96, 384 well), whereas a deep-well plate Domino is used for holding ANSI/SLAS footprint deep-well plates (up to 2 mL). Dominos are individually marked with machine-readable symbols that can be identified by the robot and can attach to each other magnetically for efficient and robust arrangement.

Because many workflows involve the use of different ontologies such as shaking, heating/cooling, magnetic bead, separation or micro-elution, connected devices, also programmable by OneLab, make up part of this workspace. In a similar manner, connected tools are used to transfer columns and microplates from one Domino to another within the workspace. An additional accessory is used for waste labware. These connected tools are placed on the device tool rack (i.e., the static arm of the robot). They are distinguished from connected devices in that tools communicate with the device they are connected to (in this case, the Andrew+ robot), whereas devices communicate directly with OneLab.

Currently, the robot can accommodate up to 11 Dominos, arranged in two rows of four4 and a row of three (see below), with devices always occupying the front row as they cannot be placed behind Dominos (**Fig. 6**).

The Andrew+ robot (**Fig. 6**), which won a new product award at its launch at SLAS 2019 in Washington, DC, was a complete redesign of the original Andrew robot, informed by the same customer input process described above.

These improvements go beyond mere performance improvements, which are to be expected when using current-generation microprocessors and microcontrollers; as an essential requirement for remote operation, they also include an embedded PC for Bluetooth communication with the electronic pipettes; the ability to use a wide range of tools such as a connected column gripper, connected microplate gripper, and connected meters and probes; and a three-dimensional environment model for trajectory and interaction with consumables. They also include a magnetic hand for the handling of generic tools and for accurate force and displacement sensing to determine dead volumes and an arm with backlash-free motors, which enables the use of multichannel pipettes for higher-throughput applications, as is often the case for genomics users.

### Tool and Connected Device Selection

At this point, it is important to highlight that unlike other liquid-handling automation on the market, the Andrew+ pipetting robot employs a full range of single and multichannel conventional electronic pipettes, dispensing volumes ranging from 0.2 µL to 10 mL. The connected electronic pipettes are actually manufactured by Sartorius in Kajaani, Finland, and they are the outcome of a collaboration in moving existing laboratory devices toward the IoT. The benefit is twofold, namely, (1) providing users with a solution whereby they can use those same pipettes independently or with the Andrew Alliance Pipette+ guided pipetting system and (2) taking advantage of an existing state-of-art and award-winning pipette. This pipette integrates among other features Bluetooth communication, enabling wireless communication with the cloud and through that bidirectional communication with OneLab, ensuring remote programming and full traceability, including data relating to the last time the pipette was calibrated.

The selection of tools is inevitably a compromise, and consideration needs to be given to the labware required, which is where flexibility is important. When developing new experimental protocols, the scale of liquid handling is initially low and then scaled up following optimization. A single aspirate and dispense cycle is the most accurate and reproducible mode of liquid handling; however, it is also the slowest. Multidispensing is used to speed things up but at the cost of accuracy. For example, an application calls for 5 µL of a reagent to be added to each well of a 96-well plate. The most accurate way of doing this would be to use a 10 µL pipette in single dispense mode, but using a 120 µL pipette in multidispense mode is much faster. The modular workspace of Andrew+ enables scaling from the use of tubes to plates by using appropriate Dominos.

In general, the choice of a connected device is solely driven by whether the ontology is required in the application or not. Like the pipettes, these too can be used as standalone devices according to user requirements, but they still benefit from remote operation by OneLab.

The different ontologies available in these connected devices impart enormous versatility to the pipetting robot workspace, enabling it to support workflows as diverse as plasmid purification, which requires a Magnet+ for magnetic bead separation, to n-Glycan analysis, which requires a Vacuum+ for solid-phase extraction prior to LC/MS.

### Systems Biology and the Drive for Common Sample Prep Solutions

To date, much effort has been expended in better understanding the pathogenicity of SARS-CoV-2. A unique aspect of the disease has been its ability to act all over the body rather than being limited to the respiratory tract, including causing strokes in otherwise healthy, younger patients.^22^ Especially dangerous is the occurrence of a cytokine storm in some patients 7 to 10 days following the onset of infection.

The data gathered thus far by the international scientific community detail the genomes and mutations of SARS-CoV-2 variants across different locations, the structure of the viral proteins, their host targets in human cells, the transcriptomics changes in infected cells, cell- or tissue-level differences in the blood or in the body of COVID-19 patients, and human genomic information from patients. It has been suggested that the only way to understand these data is by taking a systems approach that goes beyond individual actions, to connections, causes, and consequences.^23^

Ultimately, it is all about the sample, and the integration of omics data strongly depends on rigorous and consistent sample preparation protocols to reduce sample variation over short periods of time. This demands accurate tracking of each step of the sample prep conducted in the different omics workflows, including the multitude of basic yet critical liquid-handling steps, involving anything from serial dilutions to plate normalization.

Inevitably, such work is conducted in disparate locations, especially with most countries under lockdown, and the benefits of remote operation coupled with the ease of protocol sharing and method transfer can only heighten the probability of success for such vital systems-level investigations.

## Conclusions

The rapid onset of the COVID-19 pandemic has taken many research labs by complete surprise and made demands for which many researchers were unprepared. The almost global lockdown of the world’s leading economies has meant that critical public and private sector infrastructure has been managed by skeleton technical staff, with many critical project leaders mandated to work remotely, usually from a home office. Although there has been an unprecedented demand for highly accelerated development of PCR-based and serological diagnostic methods, as well as vaccines, which often take at least a decade to develop under normal circumstances, social distancing measures have made such work more challenging.

The ability to progress such research quickly demands high levels of productivity, very low error rates, and the ability to conduct experimental work remotely and collaboratively, as that is the nature of scientific research.

This implies a number of important requirements. Given the importance of sample prep and assay development, for all of the above, it also implies automation. Increasingly sophisticated automation platforms have been developed for a number of years now, but they lack a number of unique requirements demanded by the current situation, namely:

- Remote operation
- Protocol execution and adaptation without programming skills
- Ready adaptability

This is because, under normal circumstances, vendors can deploy engineers to service and retool platforms, or companies can hire/engage automation engineers or train employees on how to use software, and there usually is not the same level of urgency for accelerated research.

Andrew Alliance has developed a cloud-native software called OneLab by which protocols can be easily created and adapted, executed on a pipetting robot remotely, methods shared with other researchers worldwide, and protocol execution fully traced.
